# Validation of the Mobile App Version of the EQ-5D-5L Quality of Life Questionnaire Against the Gold Standard Paper-Based Version: Randomized Crossover Study

**DOI:** 10.2196/37303

**Published:** 2022-08-11

**Authors:** Regina J M Kamstra, André Boorsma, Tanja Krone, Robin M van Stokkum, Hannah M Eggink, Ton Peters, Wilrike J Pasman

**Affiliations:** 1 Netherlands Organization for Applied Scientific Research (TNO) Zeist Netherlands; 2 Netherlands Organization for Applied Scientific Research (TNO) Utrecht Netherlands; 3 Janssen-Cilag B V Breda Netherlands

**Keywords:** quality of life assessment, EQ-5D-5L questionnaire, mobile app, test-retest reliability, mobile phone

## Abstract

**Background:**

Study participants and patients often perceive (long) questionnaires as burdensome. In addition, paper-based questionnaires are prone to errors such as (unintentionally) skipping questions or filling in a wrong type of answer. Such errors can be prevented with the emergence of mobile questionnaire apps.

**Objective:**

This study aimed to validate an innovative way to measure the quality of life using a mobile app based on the EQ-5D-5L questionnaire. This validation study compared the EQ-5D-5L questionnaire requested by a mobile app with the gold standard paper-based version of the EQ-5D-5L.

**Methods:**

This was a randomized, crossover, and open study. The main criteria for participation were participants should be aged ≥18 years, healthy at their own discretion, in possession of a smartphone with at least Android version 4.1 or higher or iOS version 9 or higher, digitally skilled in downloading the mobile app, and able to read and answer questionnaires in Dutch. Participants were recruited by a market research company that divided them into 2 groups balanced for age, gender, and education. Each participant received a digital version of the EQ-5D-5L questionnaire via a mobile app and the EQ-5D-5L paper-based questionnaire by postal mail. In the mobile app, participants received, for 5 consecutive days, 1 question in the morning and 1 question in the afternoon; as such, all questions were asked twice (at time point 1 [App T1] and time point 2 [App T2]). The primary outcomes were the correlations between the answers (scores) of each EQ-5D-5L question answered via the mobile app compared with the paper-based questionnaire to assess convergent validity.

**Results:**

A total of 255 participants (healthy at their own discretion), 117 (45.9%) men and 138 (54.1%) women in the age range of 18 to 64 years, completed the study. To ensure randomization, the measured demographics were checked and compared between groups. To compare the results of the electronic and paper-based questionnaires, polychoric correlation analysis was performed. All questions showed a high correlation (0.64-0.92; *P*<.001) between the paper-based and the mobile app–based questions at App T1 and App T2. The scores and their variance remained similar over the questionnaires, indicating no clear difference in the answer tendency. In addition, the correlation between the 2 app-based questionnaires was high (>0.73; *P*<.001), illustrating a high test-retest reliability, indicating it to be a reliable replacement for the paper-based questionnaire.

**Conclusions:**

This study indicates that the mobile app is a valid tool for measuring the quality of life and is as reliable as the paper-based version of the EQ-5D-5L, while reducing the response burden.

## Introduction

### Background

Questionnaires are increasingly used to determine the health-related quality of life and specifically the care needs of patients; for example, as part of patient-reported outcomes [1]. Although questionnaires are perceived as an easy and noninvasive tool by researchers, study participants and patients often perceive filling out long or repeated questionnaires as burdensome. Although quality of life is often used as an outcome measure in studies, not all questionnaires used are suitable for long-term monitoring of a patient to be able to measure the course of health status over time [2,3]. Long-term monitoring of a patient’s quality of life is important for health care evaluation and may provide better insight into the actual value of interventions [4,5]. Therefore, there is a need to provide easy-to-use, patient-friendly, and valid health questionnaires. This study investigated the possibility of measuring health status in a simple and valid way using a mobile app developed by Q1.6 (Q1.6 mobile app). Using this mobile app, the response burden for participants was reduced by presenting 1 question at a time instead of requesting the complete questionnaire all at once.

Conducting paper-based questionnaires in studies is prone to errors, such as unintended skipping of questions or selecting multiple answers when 1 answer is expected [6]. In addition, data from paper-based questionnaires must be manually processed, which is time consuming, before analyses can be performed and manual entry can lead to data entry errors. With the emergence of apps for questionnaires that can be completed on smartphones, such inaccuracies can be prevented [7].

The use of smartphones and electronic devices has increased in our daily lives and in health care settings. Studies have shown that the use of smartphone app in intervention studies, for example, for self-monitoring, has become more accepted over time [8]. The use of eHealth services can lead to increased self-management of health complications [8,9]. Various patient-reported outcome measures can be queried using electronic devices [1]. The use of electronic devices is an advantage for both the individual patient and the researcher or physician: questionnaires can be answered at a convenient moment for the patient, which saves researchers’ or physicians’ and patients’ time [10]. Flexible completion could increase the frequency with which a patient is willing to complete a smartphone-based questionnaire. In addition, because of the completion of the questionnaire on the web, the data are immediately available, are stored, and can easily be compared with previously completed questionnaires. Therefore, a patient can be monitored effortlessly by the physician or researcher, and the patient does not have to make separate appointments with the physician or researcher. From a data perspective, the benefits of completing questionnaires via a smartphone app include easy to retrieve data, reliable data, and prevention of data loss due to backup systems [11]. Before a mobile questionnaire app can be deployed in a clinical setting, it is essential that this new method is validated against the traditional method [12].

In this study, a validation of a new mobile method of the well-known EQ-5D-5L questionnaire, a short questionnaire in which the quality of life of a person was examined, was performed. A comparison of the gold standard, a traditional paper version of the EQ-5D-5L questionnaire, with a digitized, mobile measurement method of (subjective) health-related quality of life via a mobile app was examined. The EQ-5D-5L questionnaire is available in various modes of administration, including smartphones [13]. Several studies have used the electronic version of the EQ-5D-5L questionnaire and indicated the electronic version valid and reliable [10,14-16]. However, the Q1.6 mobile app of the EQ-5D-5L is designed to reduce the response burden of questionnaires by presenting a single question at a time and only querying 2 questions a day rather than the whole questionnaire at once. To validate the method, the EQ-5D-5L version of the EQ-5D Health Status Questionnaire was used. This is a standardized measure of the health-related quality of life questionnaire developed by the EuroQol group [17]. EQ-5D is the most widely used tool for obtaining health outcomes from a patient’s perspective [18].

In this validation study, a comparison using correlation analysis was made between the mobile app version of the EQ-5D-5L questionnaire and the gold standard paper-based version of the EQ-5D-5L. To avoid possible bias due to time of the day (morning and afternoon), the questions in the mobile app were provided twice, referred to as time point 1 (App T1) and time point 2 (App T2), in a randomized order and differing in moment of the day (morning and afternoon).

Health-related quality of life measurement instruments must be both valid and reliable [19]. The validity of a questionnaire pertains to the degree to which the measurement reflects a construct of interest. Specifically, convergent validity is the degree to which a new scale is related to other pre-existing measures of the same construct [20]. Convergent validity between the paper, which is a pre-existing measure, and the mobile version of the EQ-5D-5L will be assessed through correlation analyses. Reliability is the consistency that the instrument measures, with test-retest reliability evaluating the consistency between 2 time points. Test-retest reliability assumes that no alterations emerge between measurements when the test is repeated and is the main aspect of reliability considered in this study [21].

Correlation analysis is the most commonly used method in validation and test-retest reliability studies and is the preferred method for this study [22]. Polychoric correlation was used for all EQ-5D-5L questions, except for the visual analog scale (VAS), because this construct uses a 5-point Likert scale [23]. Likert scales are ordinal scores, characterized by the assumption that the intervals between the scores are not equal (eg, the difference between categories 1 and 2 may not be as large as the difference between categories 2 and 3), with a limited number of possible scores. These polychoric correlations are the preferred correlational methods [24].

Owing to the longitudinal nature of the study, it was important to ensure that the differences we found were owing to differences in assessment methods and not owing to differences over time. As such, the correlation was checked between the average mobile app score (mean of both time points) and the paper score, between App T1 and the paper score, between App T2 and the paper score, and also between the 2 time points in the mobile app to study the longitudinal correlation or the test-retest validity.

### Objectives

The main objective of this study was to validate an innovative way to measure perceived health using the EQ-5D-5L questionnaire requested via the mobile app. In the validation study, a comparison using correlation analysis was made between the mobile app version of the EQ-5D-5L questionnaire and the gold standard a paper-based version of the EQ-5D-5L, in a large group of people.

In the next section, we will discuss our methods, including our study design and the statistical analyses used, followed by the results obtained by these statistical analyses and a discussion of what these results mean for the validation of the digitized Q1.6 version of the EQ-5D-5L questionnaire.

## Methods

### Study Population

Participant recruitment and data collection (questionnaires) were conducted by the research bureau MSI Advanced Customer Insights (MSI-ACI Europe BV). The participants were provided with a participant information sheet. Participants willing to participate sent in their signed and completed informed consent form digitally. To obtain a representative sample of the Dutch population, participants were selected to approximate the population distribution of age, gender, education, and place of residence. Of the 661 interested participants, 350 (52.9%) were eligible, and 261 (39.5%) eventually participated in the study. Participants were declared eligible when they met the following inclusion criteria: aged ≥18 years, healthy at their own discretion, in possession of a smartphone with at least Android version 4.1 or higher or iOS version 9 or higher, digitally skilled enough to download the mobile app, and able to read and answer questionnaires in Dutch. Participants who completed the study received an incentive (digital bol.com store voucher) of €10 (US $12).

### Ethics Approval

The study plan was approved by the Internal Review Board of TNO (Netherlands Organisation for Applied Scientific Research) on February 12, 2021 (number 2021-009). The study was conducted in accordance with the current assembly (64th) of the Declaration of Helsinki (Brazil, October 2008), which was updated by the WMA (World Medical Association) General Assembly in 2013. The study was conducted in March 2021 and completed in 4 weeks.

### Study Design

**Figure 1:**
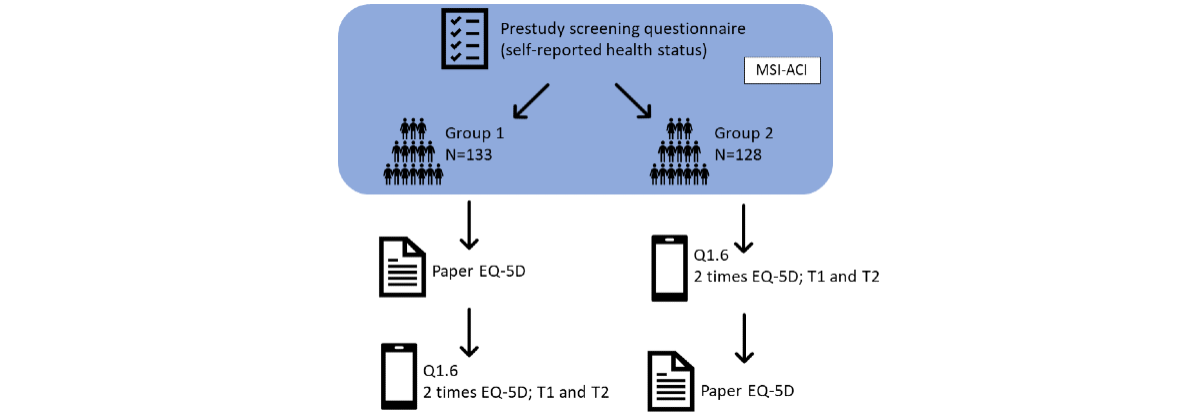
Study design. The figure shows the order of paper-based version and the mobile app questionnaires for groups 1 and 2. With exception of a visual analog scale score, questions of the EQ-5D-5L were asked twice via the mobile app. A single question was requested once in the morning and once in the afternoon on 5 consecutive days. MSI-ACI: MSI Advanced Customer Insights; T1: time point 1 (app question answered first time); T2: time point 2 (same app question answered second time).

This was a randomized, crossover, and open study. Eligible participants (n=261) were assigned to groups 1 or 2. A total of 50.9% (133/261) of participants were assigned to group 1. The participants in group 1 first received a paper-based questionnaire. They were given a maximum of 1 week to complete the questionnaire on paper and return it by postal mail. Thereafter, the participants were informed by email to start with the mobile app. A total of 49% (128/261) of participants were placed in group 2. Participants in group 2 first completed the mobile app questionnaire, followed by the paper-based questionnaire, which was sent by postal mail. Of the 261 participants, 255 (97.7%) participants completed both the paper-based version of the EQ-5D-5L and the mobile app version. A schematic overview of the study design is shown in Figure 1.

### The EQ-5D Paper-Based Questionnaire

The EQ-5D is a standardized measure of health-related quality of life questionnaire developed by EuroQol. The EQ-5D is the most widely used tool for obtaining quality of life from a patient’s perspective [18]. The EQ-5D is a descriptive instrument that focuses on five dimensions of health: mobility, self-care, usual activities, pain or discomfort, and anxiety or depression. Two versions of the EQ-5D are available: EQ-5D-5L (the 5-level version, each domain consisting of 5 levels; “1=no problems, 2=slight problems, 3=moderate problems, 4=severe problems, and 5=extreme problems”) and EQ-5D-3L (the 3-level version, each domain consisting of 3 levels; “1=no problems, 2=some problems, and 3=extreme problems”). Lower scores indicate better quality of life. The EQ-5D-5L questionnaire was used in this study. The second part of the paper-based questionnaire comprises a standard vertical 20-cm line representing a scale from 0 to 100, also known as the VAS. The respondents are asked to score their current health status on the scale and write the corresponding number in an adjacent box. A higher VAS score represents a better perceived health status.

### The Q1.6 Mobile Phone App

The company Q1.6 developed a mobile app that prompts a participant to answer a short question about their health twice a day. For this study, the EQ-5D-5L was configured in the Q1.6 app (Multimedia Appendix 1). The participants were notified to download the mobile app to their personal mobile phones. For 5 consecutive days, the participants received notifications for 2 EQ-5D-5L questions per day. A single question was posed once in the morning and once in the afternoon. The EQ-5D-5L questions were randomized to be presented in the morning or afternoon to increase generalizability. On the fifth day, the participants also had to complete the VAS score. In total, all questions were asked twice spread over 5 consecutive days, with the exception of the VAS score, which was asked only once. Notifications prompting the participant to answer a question were sent to the participants’ smartphones between 8 AM and 10:30 PM and closed automatically. Participants in possession of an iPhone received a notification every consecutive hour until the question was answered. On Android, questions were prompted when a user unlocked their phone until the question was answered.

Subjective evaluation of the mobile app was requested via an evaluation questionnaire in the mobile app to gain insight into the usability of the mobile app.

### Sample Size

The number of participants needed in the study was based on a power calculation assuming a correlation of 0.7 with an α of .05 and a power of 0.90. To allow for the fact that any correlation is inherent in health measurements in general, no test against finding no correlation was performed, but the estimation of power was made to distinguish between the expected correlation of 0.7 and a more general correlation of 0.5, which may be found in any 2 health-related questionnaires. On the basis of G*power calculation [25], we required 200 participants to validate the mobile app questionnaire using these parameters.

### Statistical Analyses

Descriptive variables were compared between the 2 experimental groups (ie, group 1: first paper-based EQ-5D-5L questionnaire or group 2: first mobile app questionnaire) to check whether randomization had achieved a good spread of baseline variables across the groups in the study. Participants missing >1 question in the mobile app (5, 1.9%) were removed from the analysis to have as many completed questionnaires as possible for validation (approximately 200 completed on paper and via the mobile app).

To compare the paper- and app-based questionnaires, polychoric correlation analyses were used for all EQ-5D-5L questions, except for the VAS scale, as the questions were answered using a 5-point Likert scale [23]. For these calculations, the package Lavaan (version 0.6-9) in R (designed by Robert Gentleman and Ross Ihaka and developed by the R Core Team) was used [26]. Pearson correlation was estimated for the VAS. For the main outcome, that is, the correlation between paper and the mobile app, the average score over 2 days in the mobile app was correlated with the observed score from the paper version. The answers given the first time a question appeared in the mobile app were combined at time point 1 (App T1); the answers provided during the second time were combined at time point 2 (App T2). In addition, the correlations between the observed score on paper and App T1, and the observed score on paper and App T2 were calculated, as presented in Multimedia Appendix 2.

To study the longitudinal correlation or test-retest reliability, we examined the test-retest correlation of the 2 time points using the mobile app (App T1 vs App T2) and the validity assessment (paper vs digital [the mobile app]) through the polychoric correlation. With similar correlation coefficients (eg, within a few points of each other), we can assume that both methods measure the same. Furthermore, paired *t* tests (2-tailed) were used to check whether participants consistently scored higher or lower on paper or on the mobile app (Multimedia Appendix 3).

Data are presented as mean (SD). A *P* value of <.05 was considered statistically significant. Correlation values of 0.0 to 0.3 indicate weak agreement, 0.3 to 0.5 indicate mediocre agreement, 0.5 to 0.7 indicate strong agreement, and >0.7 indicate very strong agreement [27]. Box plots are presented for descriptive analyses of the usability of the mobile app.

## Results

### Descriptive Results of Study Participants

A final data sample of 255 participants (117 males—group 1: 54, 43%; group 2: 63, 49% and 138 females—group 1: 73, 57%; group 2: 65, 51%), with an age of ≥18 years and no more than one observation missing, were included in the analysis. Although there was no maximum age set for inclusion of participants, the maximum age of respondents was 64 years. In total, 56.5% (144/255) of the participants were highly educated and had obtained at least a bachelor’s degree. The demographic data of the participants are presented in Table 1. Characteristics are listed in total and per condition (group 1: paper first; group 2: mobile app first). The distribution of the demographic characteristics between the groups was consistent. Group 2 appeared to have slightly more people aged >50 years (52/128, 40.6% vs 35/127, 27.6% in group 1) and people living in the eastern part of the Netherlands (39/128, 30.5%) than in group 1 (24/127, 18.9%).

**Table 1:** Baseline characteristics of the participants (N=255) who completed the study in total and the representation in the group.

Parameter		Participants, n (%)	Group 1 (paper first), n (%)	Group 2 (mobile app first), n (%)
**Gender**				
	Man	117 (45.9)	54 (42.5)	63 (49.2)
	Woman	138 (54.1)	73 (57.4)	65 (50.7)
**Age (years)**				
	18-34	88 (34.5)	48 (37.8)	40 (31.3)
	35-49	80 (31.4)	44 (34.6)	36 (28.1)
	50-64	87 (34.1)	35 (27.6)	52 (40.6)
**Education^a^**				
	Low	10 (3.9)	4 (3.1)	6 (4.7)
	Middle	101 (39.6)	46 (36.2)	55 (43)
	High	144 (56.5)	77 (60.6)	67 (52.3)
**Region^b^**				
	Cities	27 (10.6)	15 (11.8)	12 (9.4)
	North	25 (9.8)	11 (8.7)	14 (10.9)
	East	63 (24.7)	24 (18.9)	39 (30.5)
	South	56 (22)	26 (20.5)	30 (23.4)
	West	84 (32.9)	51 (40.2)	33 (25.8)

^a^Participants who only attended primary education, prevocational secondary education (VMBO or LBO) were defined as less educated. Participants with a secondary school (preparatory vocational secondary education [mavo], senior general secondary education [havo], or university preparatory education [vwo]) or senior secondary vocational education and training (MBO) degree were defined as middle educated. Participants with at least a bachelor’s degree in higher professional or university education were categorized as having a higher education.

^b^Participants were recruited from all over the Netherlands. An overview of participants living near big cities and region of the Netherlands is presented.

### Descriptive Results by Domain

The average scores of the different variables on paper, App T1 (first time question in the mobile app) and App T2 (second time question in the mobile app) are presented in Table 2. With the exception of the VAS score (range 0-100), a lower score indicates a better quality of life (range 1-5). As can be seen, the group generally considers themselves healthy and scores between 1 and 2 on the five domains (mobility, self-care, usual activities, pain or discomfort, and anxiety or depression) across the different media (paper vs the mobile app). The mean VAS score for paper administration was 77.7 (SD 14.3), and the mean VAS score for the mobile app was 78.7 (SD 15.8). The scores and their variability remained similar over the questionnaires, indicating no clear difference in the answer tendency. Within self-care, a very low variance and overall, a low score were observed (paper average 1.1, SD 0.4; mobile app average 1.1, SD 0.3). Multimedia Appendix 3 presents 2-tailed *t* tests pertaining to the difference in responses on the paper and mobile app versions.

**Table 2:** Average score per EQ-5D-5L question.

Variable	Paper, mean (SD)	App, mean (SD)	App T1^a^, mean (SD)	App T2^b^, mean (SD)
Visual analog scale score	77.705 (14.324)	N/A^c^	N/A	78.697 (15.751)
Anxiety or depression	1.596 (0.845)	1.542 (0.782)	1.556 (0.798)	1.522 (0.832)
Pain or discomfort	1.659 (0.831)	1.587 (0.716)	1.616 (0.780)	1.578 (0.767)
Usual activities	1.467 (0.730)	1.437 (0.713)	1.410 (0.747)	1.451 (0.735)
Self-care	1.110 (0.439)	1.086 (0.332)	1.078 (0.335)	1.093 (0.354)
Mobility	1.247 (0.619)	1.247 (0.558)	1.235 (0.579)	1.240 (0.556)

^a^App time point 1 (App T1) was the first time the question was answered.

^b^App time point 2 (App T2) was the second time that the same question was answered.

^c^N/A: not applicable (via the mobile app, each question of the EQ-5D-5L, with the exception of the visual analog scale score, was requested twice).

### The Mobile App—Retest Results

The EQ-5D-5L questions in the mobile app were answered twice. Correlations between App T1 and App T2 were high (range 0.73-0.9; *P*<.001; Table 3), with the highest correlation observed for self-care and the lowest for anxiety or depression and mobility. This indicates high test-retest reliability across all domains. Compared with the correlations between methods (Table 4), the differences were small (range 0.19-0.02). The CIs overlapped with a notable correlation with self-care. However, this may be because of the very low variance within self-care (Table 2), causing a small difference in answers and generating a disproportionally large influence.

**Table 3:** Polychoric correlations between app time point 1 (App T1) and time point 2 (App T2).

App T1 vs App T2	Correlation	SE	*z* score	*P* value	Range (lower-upper)
Anxiety or depression	0.729	0.029	24.838	<.001	0.671-0.786
Pain or discomfort	0.744	0.028	26.640	<.001	0.689-0.799
Usual activities	0.786	0.024	32.855	<.001	0.739-0.833
Self-care	0.897	0.012	73.058	<.001	0.873-0.921
Mobility	0.730	0.029	24.906	<.001	0.672-0.787

**Table 4:** Polychoric correlations between EQ-5D-5L paper and EQ-5D-5L mobile app (average App T1 and App T2) version.

Digital variable versus paper	Correlation	SE	*z* score	*P* value	Range (lower-upper)
VAS^a^_digital	0.636	0.030	20.876	<.001	0.576-0.696
Anxiety or depression	0.791	0.122	6.496	<.001	0.553-1.030
Pain or discomfort	0.824	0.121	6.783	<.001	0.586-1.062
Usual activities	0.812	0.122	6.657	<.001	0.573-1.052
Self-care	0.880	0.124	7.083	<.001	0.636-1.123
Mobility	0.915	0.121	7.545	<.001	0.677-1.152

^a^VAS: visual analog scale.

### Correlations Paper-Based EQ-5D-5L Questionnaire Versus the Mobile App

Table 4 displays the results of the polychoric correlations, showing high relations between the different scores on paper and in the mobile app (average App T1 and App T2) across the 5 domains (>0.79). It is difficult to set a cutoff value based on a variety of assumptions and indicators. However, as all correlations between the paper and digital questions are above 0.79, they indicate a very strong validity for repeated questions about anxiety or depression, pain or discomfort, usual activities, mobility, and self-care. Mobility showed the strongest correlation (0.92; *P*<.001). In addition, the correlations of the EQ-5D domains paper versus app were in the same range as the test-retest reliability results. The estimated correlation of the VAS scores was lower (0.64; *P*<.001) compared with the questions on the 5 domains (>0.79; *P*<.001).

The polychoric correlations between paper and App T1 and between paper and App T2 are presented in the Multimedia Appendix 2. Lower correlations were observed between paper and App T1 (>0.64) and paper and App T2 (>0.65), compared with the correlations between paper and the average score of the mobile app (>0.79) and compared with the correlations between App T1 and App T2. Using paired 2-tailed *t* tests, no significant differences between the average mobile app score and the observed paper score were found (Multimedia Appendix 3) or between App T1 and App T2 (Multimedia Appendix 4). Consistently higher or lower scoring of paper compared with the mobile app was also not observed. A strong agreement between the paper and the mobile app was observed (>0.64), with no structurally higher scores on paper or the mobile app.

The slight difference in correlation strength between the correlations between App T1 and App T2 (Table 3) and the correlations found between paper and the mobile app (Table 4) may be because of the effect of time (between the 2 moments of answering in the mobile app) and not the difference in media (paper vs the mobile app). This is because the perceived quality of life changes slightly over the course of a day and between days, even in a healthy population [28].

### Subjective Evaluation of the Mobile App

**Figure 2:**
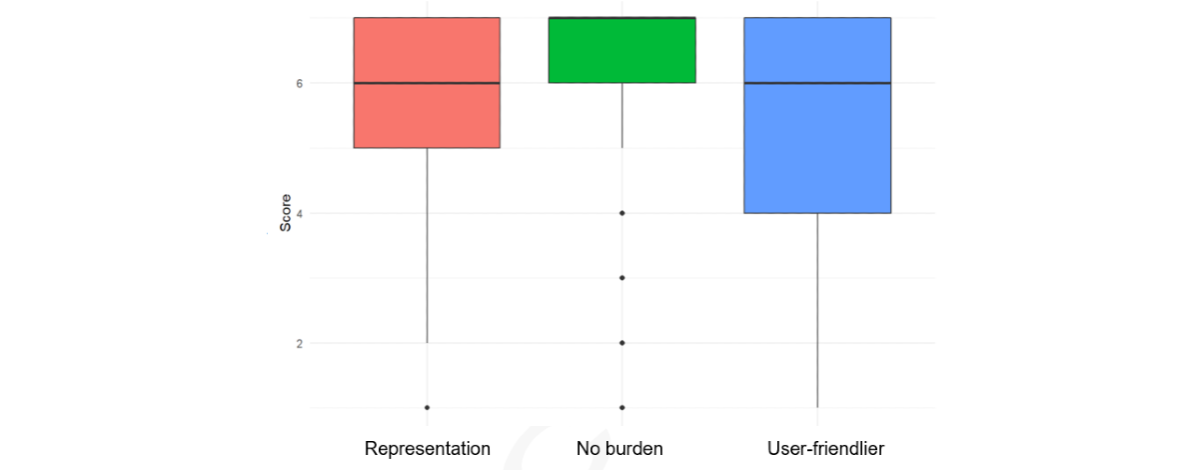
Usability of the mobile app, where a higher score indicates higher agreement with the question posed, the questions being, “The app provides a good representation of how I feel,” “It is no burden to answer this questionnaire quarterly,” and “The app is more user-friendly than the paper-based questionnaire.”.

A short evaluation questionnaire was requested via the app to gain insight into its usability. Figure 2 shows the usability on three aspects: “the app provides a good representation of how I feel,” “it is no burden to answer this questionnaire quarterly,” and “the app is more user-friendly than the paper-based questionnaire.” The scores ranged from 1 to 7, with a higher score indicating that the participants strongly agreed with the statement.

Most (197/261, 75.5%) participants indicated that the mobile app provided a good representation of how they felt. Furthermore, the participants perceived no burden to answer this questionnaire quarterly via the mobile app. In addition, the mobile app was perceived as more user-friendly than the paper-based questionnaire. However, some (42/261, 16.1%) participants mentioned in their free-text comments that user-friendliness could be improved in some ways. For example, a couple of participants mentioned that the notification or icon of the mobile app always remained at the top left on their phone screen, which was experienced as unpleasant.

## Discussion

### Principal Findings

This study aimed to validate an innovative way to measure health-related quality of life using a mobile app based on the EQ-5D-5L questionnaire that may replace the paper version of the questionnaire. The mobile app prompts a participant to answer a single question of the EQ-5D-5L questionnaire twice a day, once in the morning and once in the afternoon. In total, all questions were asked twice, spread over 5 consecutive days. This study was designed to compare the scoring in the mobile app to the gold standard paper-based version of the EQ-5D-5L.

This study showed high correlations, of over 0.79 (*P*<.001), for all 5 questions between the paper-based EQ-5D-5L questionnaire and the mobile app questionnaire (averaged App T1 and App T2). When comparing the paper-based version separately with the 2 time points App T1 and App T2 (Multimedia Appendix 2), the correlations were slightly lower. Depending on the domain (eg, mobility, self-care, usual activities, pain or discomfort, and anxiety or depression), the correlations between the paper-based version and App T1 or App T2 were higher. For all domains, the correlation of the mean of both entries was higher than that for one entry, regardless of whether it is App T1 or App T2. Averaging the 2 scores may have eliminated some variability, potentially increasing the correlation. Nevertheless, because of the high correlations for App T1 (>0.64) and App T2 (>0.65), completing the questionnaire once via the mobile app seems to be sufficient, considering its high retest reliability. In addition, no significant differences between the paper score and the mobile app were observed, indicating that the scores were not structurally higher for either paper or the mobile app. Regarding test-retest reliability, high correlations were found between the App T1 and App T2 scores (>0.73). Both the high correlation between the paper version and the mobile app questionnaire, along with the high correlation of the in-between comparison of the mobile app time points (App T1 vs App T2), make the new mobile version of the EQ-5D-5L questionnaire a reliable replacement for the paper-based method.

A review [12] demonstrated the equivalence of electronic and paper-based patient-reported outcome measures. Equivalence was observed in 43 studies. However, 2 studies did not find equivalence, and 10 studies had no clear conclusions. Furthermore, Jiang et al [16] criticized the web-based method in comparison with the face-to-face method. Therefore, validation of the new smartphone method compared with the gold standard is important before using the new method in a health care setting.

Belisario et al [7] compared the responses to questionnaires using a mobile app with other methods (eg, paper, laptop, and web based). No major differences in using the app or “other methods” were observed. This study used a threshold correlation of >0.6, which is in line with our results, and we found correlations of >0.64.

Mulhern et al [10] executed a comparable study with the EQ-5D-5L questionnaire requested on paper and via a mobile phone app. In this study, a higher response rate was observed for mobile phone questionnaires. However, their study used a parallel design; the participants completed only one administration method. In comparison, in this study, participants completed both the paper-based and mobile app versions of the EQ-5D-5L. This study design is recommended to have confirmative evidence for equivalence [29], because the same participants undergo both methods. In a study by Lundy et al [30], the EQ-5D-5L paper version was examined using different devices (handheld, tablet, interactive voice response, and web). They found substantial evidence supporting the measurement equivalence of the different modes of data collection (paper format and screen-based and phone-based formats of the EQ-5D-5L provided) [30]. Evidence that this is not the case for the current questionnaire was found in the study by Kim et al [31]. They showed high correlations for the International Prostate Symptom Score questionnaire requested via an app and on paper. Furthermore, Bellamy et al [32] found high correlations between a paper-based and mobile-based scores for osteoarthritis. Different studies [11,33,34] showed a similar response to a questionnaire requested via an app and a paper-based version. These results support the validity of the EQ-5D-5L mobile app questionnaire used in this study in healthy volunteers.

An attribute of the questionnaire itself in a healthy population, such as this, is the low variance. Most people answered that they did not have any daily issues on each of the Likert scale questions. Therefore, polychoric correlation was applied, because this method is more sensitive to ordinal data with low variance. The reliability and validity of the mobile app in unhealthy people should be tested in a follow-up study, because the variance in the domains may be higher, which could affect the correlations between the methods.

We found a lower correlation for the VAS score (0.62; *P*<.001) than for the 5 domain questions between the 2 methods. This may be because of the greater sensitivity to the daily differences in this score, as the scale runs from 0 to 100 instead of from 1 to 5 as the domain questions (continuous vs categorical data). However, most individuals (204/255, 80%) scored >70, leaving an effective score range of 70 to 100. This means that an individual who scores 74 today and 75 tomorrow has an increase, whereas an individual who scores 74 today and 72 tomorrow has a decrease in score, although both deviations may be a nonnoticable difference for the individual. When asked about categories, this would be the same category, and no deviations would be depicted.

Earlier studies showed a low yet positive correlation between consecutive measurement moments within persons for the EQ-5D VAS of 0.21 [35], indicating that a lagged correlation of 0.62 seems reasonable for a lagged relationship between individuals. In this study, there were several days between completing the questionnaire on paper and via the mobile app, which might have caused differences in the VAS scores.

Furthermore, the display length of the mobile device could affect the 0 to 100 scores differently compared with the paper length, resulting in more variation. However, other studies showed no effect of differences in device length on VAS scores; they were all similar even if the screen was half the size, meaning that the VAS itself may be a constant measure [36].

### Strengths and Limitations

This study has some limitations. In this study, most (144/255, 56.5%) participants were highly educated, and only 4% (10/255) of the participants had a low education level. Therefore, it is not yet known whether this mobile app is usable for lower educated people and whether education level affects the validity of the paper-app comparison. Furthermore, the usability for older people is not known, because the maximum age in the study was 64 years. In addition, although the app-based questionnaire is relatively simple, it does not test which minimal digital skills are needed for the use of the mobile app. The participants in the study were digitally skilled (as part of the inclusion criteria) and indicated that the questionnaire app was user-friendly (Figure 2). In addition, the mobile app was only tested with people who stated to be healthy at their own discretion, which may have caused low variance in scores.

Most eHealth app studies have nonrepresentative populations [37,38]. This indicates that there may have been a selection bias during the recruitment of people for this type of study. Nicholl et al [37] also reported that participants were predominantly female, White, well educated, and middle aged, and thus the wider applicability of digital self-management interventions remains uncertain. This is important for the usability of the at-home tests. However, this may not be applicable to the general population.

Despite these limitations, a good representation of the population with sufficient spread of age, gender, and people living in the Netherlands was obtained. Furthermore, the number of participants was sufficient for validation (calculated necessary: 200; completed the study: 255). Another strength of this study was the testing period of the mobile app before the start of the study. Before the study began, the mobile app was optimized using feedback from the testers.

### Future Studies

Only healthy participants were included in this study. It is recommended to test the app in distinct groups of patients to gain insight into whether the perception of quality of life in diseased people can be measured with the mobile app as well.

An app should be attractive through pictures and should not be textual. Positive feedback and rewarding also play key roles in eHealth interventions [39]. The long-term use of and compliance with mobile apps require special attention. Recommendations for the app can be provided by using an extensive usability questionnaire. AB testing, which allows for the comparison of different variations of the mobile app, may be used to improve mobile app usability. Aesthetics (attractiveness), utility (relevance), and usability play vital roles in eHealth.

In addition, the study population consisted mainly of highly educated individuals. It is recommended that the mobile app be tested in a low socioeconomic group of people too. It is also worthwhile to validate the mobile app for use by older people. Older people are often less comfortable using mobile apps, and information about quality of life may be even more relevant for this group [40]. Another recommendation is to determine the use of the mobile app in long-term monitoring; for example, as a follow-up tool during or after treatment [11,31,34].

In addition, there is another instrument for scoring perceived health, the health monitor. The health monitor is inspired by the self-anchoring scale, also known as Cantril Ladder, which uses 10 steps to stress one’s health [41], combined with a short questionnaire. The health monitor is used to measure a person’s perceived acceptance and control of their illness or well-being. Future research should investigate whether the health monitor is comparable with the EQ-5D and could be used as a future quality of life tool. Furthermore, the next level of nonobtrusive measurement of quality of life could be achieved by means of digital phenotyping. Digital phenotyping is the quantification of a particular human behavior using data from personal digital devices or wearables. In the near future, data such as proximity to other devices using Bluetooth, estimation of activity, or detection of voice could be applied as robust, continuous nonintrusive proxies of aspects of quality of life [42].

### Conclusions

In this study, high correlations between the questionnaire requested via the mobile app and on paper were observed. This indicates that the mobile app is valid for use and is as reliable as the paper-based version of the EQ-5D-5L. With the widespread use of mobile phones, the mobile app is potentially valuable for perceiving a patient’s health in a simple and valid way, as an alternative to the paper-based EQ-5D-5L questionnaire. The mobile app could reduce the current burden and errors with the use of questionnaires, such as skipping questions, giving more answers than required, and data entry errors. In addition, mobile app data will be immediately available and stored and can easily be compared with previously completed questionnaires. However, more research is required to establish the use of mobile apps for consecutive monitoring of various user groups.

## Appendix

Multimedia Appendix 1 Example (in Dutch) of EQ-5D-5L question (mobility) programmed in the Q1.6 app.

Multimedia Appendix 2 Polychoric correlations between EQ-5D-5L paper and EQ-5D-5L App T1 and App T2.

Multimedia Appendix 3 Paired 2-tailed *t* test for mean difference in digital versus paper measurements.

Multimedia Appendix 4 Paired 2-tailed *t* test for mean difference in App T1 versus App T2.

## Abbreviations

VAS: visual analog scale
WMA: World Medical Association
