# Supplementary figures and images for: Validation of the Mobile App Version of the EQ-5D-5L Quality of Life Questionnaire Against the Gold Standard Paper-Based Version: Randomized Crossover Study
Source: JMIR Form Res. 2022 Aug 11;6(8):e37303. doi: 10.2196/37303 (PMC9412727; doi:10.2196/37303)

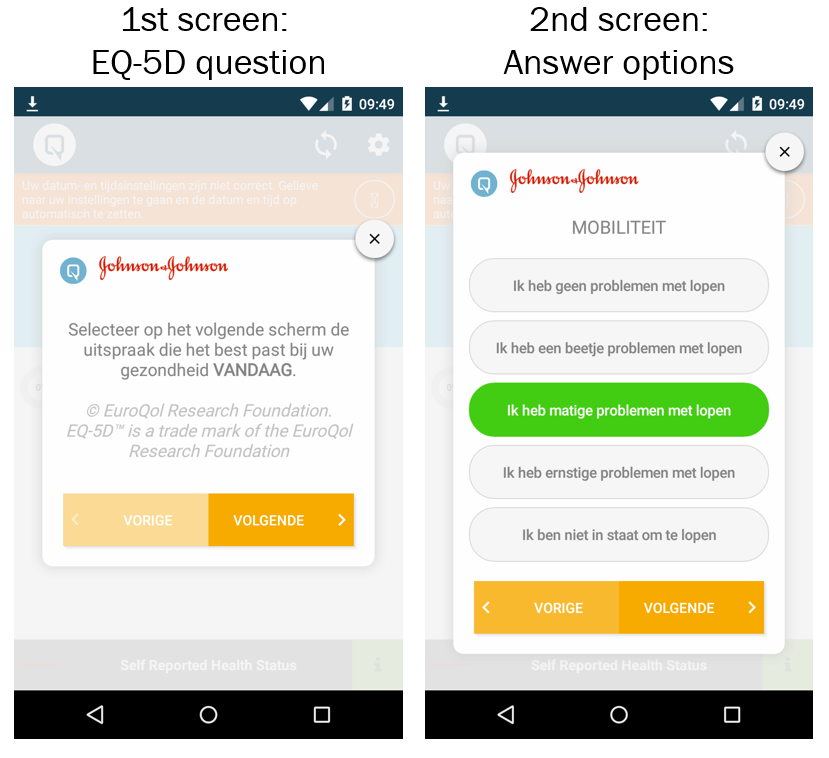

Supplement: Multimedia Appendix 1 [file formative_v6i8e37303_app1.png]

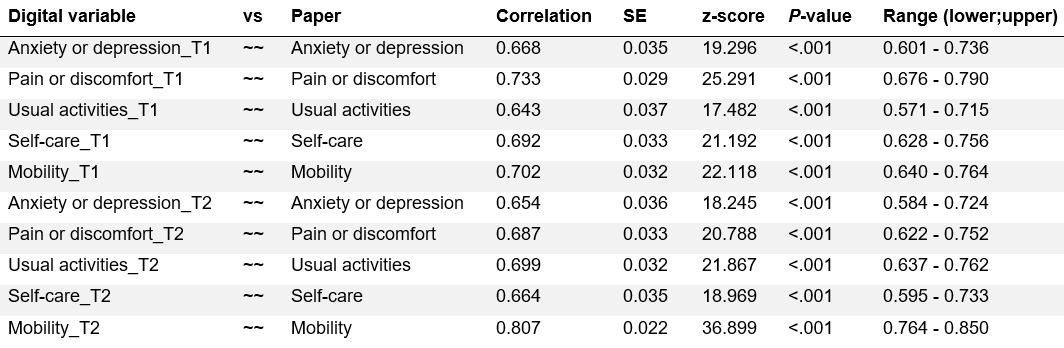

Supplement: Multimedia Appendix 2 [file formative_v6i8e37303_app2.png]

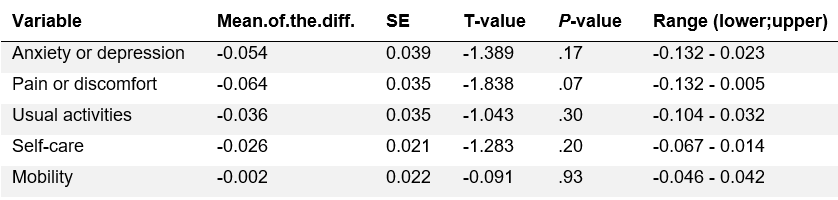

Supplement: Multimedia Appendix 3 [file formative_v6i8e37303_app3.png]

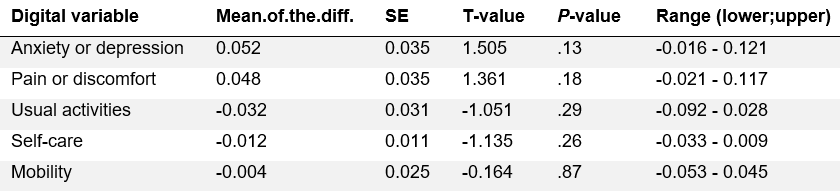

Supplement: Multimedia Appendix 4 [file formative_v6i8e37303_app4.png]
